# Spatial and Temporal Gene Expression Differences in Core and Periinfarct Areas in Experimental Stroke: A Microarray Analysis

**DOI:** 10.1371/journal.pone.0052121

**Published:** 2012-12-17

**Authors:** Jaime Ramos-Cejudo, María Gutiérrez-Fernández, Berta Rodríguez-Frutos, Mercedes Expósito Alcaide, Fátima Sánchez-Cabo, Ana Dopazo, Exuperio Díez–Tejedor

**Affiliations:** 1 Department of Neurology and Stroke Centre, Neuroscience and Cerebrovascular Research Laboratory, La Paz University Hospital, Neuroscience Area of IdiPAZ (Health Research Institute), Autónoma University of Madrid, Madrid, Spain; 2 Bioinformatics Unit, Centro Nacional de Investigaciones Cardiovasculares (CNIC), Madrid, Spain; 3 Genomics Unit, Centro Nacional de Investigaciones Cardiovasculares (CNIC), Madrid, Spain; Institute of Neurology (Edinger-Institute), Germany

## Abstract

**Background:**

A large number of genes are regulated to promote brain repair following stroke. The thorough analysis of this process can help identify new markers and develop therapeutic strategies. This study analyzes gene expression following experimental stroke.

**Methodology/Principal Findings:**

A microarray study of gene expression in the core, periinfarct and contralateral cortex was performed in adult Sprague-Dawley rats (n = 60) after 24 hours (acute phase) or 3 days (delayed stage) of permanent middle cerebral artery (MCA) occlusion. Independent qRT-PCR validation (n = 12) was performed for 22 of the genes. Functional data were evaluated by Ingenuity Pathway Analysis. The number of genes differentially expressed was 2,612 (24 h) and 5,717 (3 d) in the core; and 3,505 (24 h) and 1,686 (3 d) in the periinfarct area (logFC>|1|; adjP<0.05). Expression of many neurovascular unit development genes was altered at 24 h and 3 d including *HES2*, *OLIG2*, *LINGO1* and *NOGO-A*; chemokines like *CXCL1* and *CXCL12*, stress-response genes like *HIF-1A*, and trophic factors like *BDNF* or *BMP4*. Nearly half of the detected genes (43%) had not been associated with stroke previously.

**Conclusions:**

This comprehensive study of gene regulation in the core and periinfarct areas at different times following permanent MCA occlusion provides new data that can be helpful in translational research.

## Introduction

Clinical management of stroke, a major cause of death and disability all over the world, still requires effective new therapeutic targets and markers. Most trials based on neural protection or immune-suppression have failed or obtained poor results, and effort and interest are now dedicated to the development of new strategies that could enhance naturally-occurring endogenous self-repair processes [1]. Genetic research has constantly revealed that plasticity and resilience exist in both the developing and the adult brain [2] and great efforts have gone into unraveling the molecular events underlying reorganization and plasticity following stroke damage [3], [4], [5]. While it is accepted that such processes become activated, most are still not completely identified [6], [7], [8].

Gene expression levels can vary in different brain areas subjected to ischemic conditions and also across the different stages of the ischemic cascade. In this sense, the broad view of regulation provided by microarray analysis can offer new insight into mechanisms of stroke and repair [9]. Using this technique to focus on the periinfarct area 24 h after transitory focal cerebral ischemia, earlier authors identified potential new tissue repair and plasticity targets as well as multiple transcripts not previously associated with stroke [10]. Most gene expression studies to date have focused on transitory ischemia, but transcriptional events after permanent occlusion - a common condition in stroke patients - has received little attention. Indeed, the mechanisms of infarct evolution and the responsiveness of lesions are not necessarily equivalent from one model to another [11] and therefore, the information provided after analyzing responsiveness to permanent occlusion can also be useful.

In the last decade the emerging concept of penumbra has gained importance in terms of metabolism and cellular signals and is now considered a diagnostic and biochemical target as well as the starting point for studying brain plasticity [12]. Classically defined as hypoperfused and functionally compromised tissue surrounding the ischemic core of the lesion, both the definition of penumbra and its clinical implications have constantly evolved. After volumetric analysis, including quantitative ADC and CBF voxel compartmental analysis, of temporal evolution after permanent middle cerebral artery occlusion, it was recently reported that the distribution pattern of the penumbra was rather complex and did not follow the classically-described pattern surrounding a central core [13]. It has also recently been reported [14] that metabolic status and spreading depolarization of periinfarct neurons is critical for the recruitment of these neurons into the ischemic core over time. Thus, not only blood flow or glucose/O2 deprivation–induced stress but also metabolic status have been shown to be critical. Understanding the differences between the core of the lesion and the surrounding compromised periinfarct (PI) areas could well offer new targets and strategies for the treatment of stroke. Indeed, recent efforts have been made to analyze the metabolic evolution of both areas after both transitory and permanent occlusion [15].

From this perspective, the manner in which gene expression is modulated in different areas of the brain after permanent ischemia has not yet been studied with microarrays and such information could be of great interest.

The present study has used microarray technology to identify thousands of genes whose expression is significantly altered in both the core and the periinfarct areas at both 24 h and 3 d after permanent middle cerebral artery occlusion (pMCAo) in rats.

## Results

### Early and Delayed Transcriptional Regulation Following Cerebral Infarct

**Figure 1:**
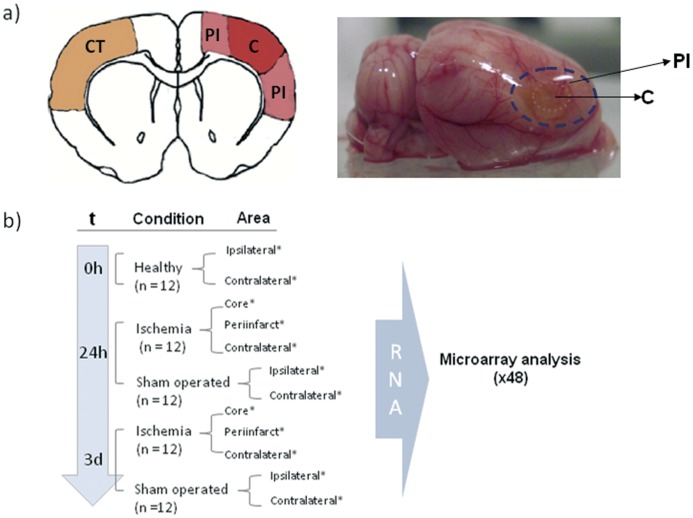
The pMCAO model produced cortical infarcts where core, periinfarct, and contralateral areas were isolated for RNA extraction. Sprague-Dawley rats were subjected to permanent middle cerebral artery occlusion with a 9-0 suture for 24 h or 3 d (a) Schematic drawing of brain areas used for tissue sampling and RNA extraction. (b) Panel shows workflow and study groups used (n = 12 animals/condition). * = 4 RNA pools/condition (1 pool = 3 animals = 1 microarray). (CT: Contralateral cortex; PI: Periinfarct; C: Core).

Tissue samples were obtained from different areas of the rat brain (core, periinfarct and contralateral cortex) at 24 h (early stage) or 3 d (delayed injury) after permanent middle cerebral artery occlusion (pMCAO). We designed a protocol to minimize variability and improve the reproducibility of the results. For microarray analysis a total of 60 animals (n = 12 for each condition; 5 groups) and 48 microarrays were used (4 microarrays/condition; RNA samples from 3 independent animals were pooled in each microarray) (**Figure 1**); results obtained from ischemic and sham-operated animals were compared with results from healthy animals. We used healthy animals as the control group as we did not find significant differences between sham-operated and healthy animals. In order to gain in simplicity and since bloodflow in the contralateral cortex is also reduced in the 3VO model of pMCAO and thus the tissue cannot be considered entirely healthywe did no further analysis of the contralateral hemisphere.

**Figure 2:**
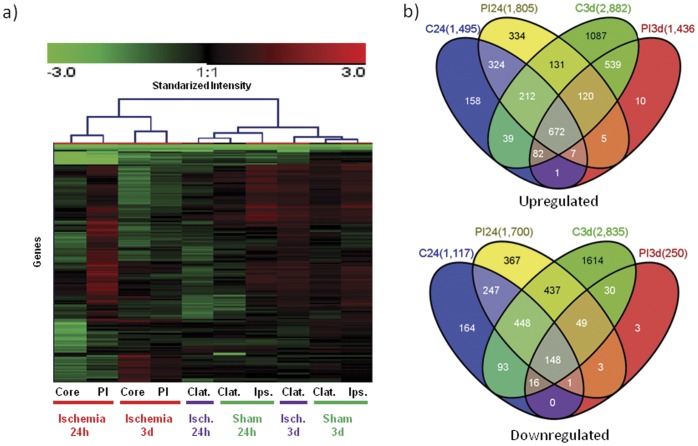
Focal cerebral ischemia induces early and delayed gene regulation with different patterns in core and periinfarct areas. RNA was isolated (Qiagen RNeasy) and hybridized to Agilent chips (4×44 K). (a) Upper part of the Heat map of the genes with an at least 2-fold change in expression in at least one condition after 24 h and 3 d of permanent cerebral infarct vs. levels in healthy samples. Each experimental condition is given at the bottom. PI = Periinfarct area; Clat = Contralateral cortex; Ips = Ipsilateral area. Vertical “Y” axis shows individual genes. Red = Up; Black = no change; Green = down. (b) Venn diagrams of upregulated and downregulated genes showing differences between core (C) and periinfarct (PI) at 24 h and 3 days of ischemia compared with healthy individuals (logFC = |1|; *P*<0.05).

The analyzed brain regions responded with different spatial and temporal patterns after the ischemic insult. Hierarchical clustering of the genes with changes in expression in at least 1 condition vs. healthy samples (**Figure 2A**) showed that brain areas subjected to experimental ischemia (core and PI) grouped together and were differentiated from the other conditions (Contralateral cortex, or Sham-operated). Only 165 genes showed an adjusted p-value of less than 0.05 for the comparison between sham-operated and healthy rats. A large number of genes were differentially expressed in the infarct core (2,612) 24 h after permanent ischemia compared with the same ipsilateral regions from healthy animals (**Table 1**). A similar situation occurred in the PI area, where 3,505 genes changed their expression levels at 24 h (1,805 up- and 1,700 down-regulated) with logFC’s greater than 5 in many cases. In another independent subgroup of animals analyzed after 3 d the situation was reversed with 1,686 genes regulated in PI and a total of 5,717 genes in the core compared with gene expression in healthy animals.

**Table 1:** Number of genes differentially regulated at 24 hours and 3 days after pMCAO.

Total regulated probes	24 h		3 d		24 h	3 d
	Core	PI	Core	PI	Core vs PI	Core vs PI
Total	**2,612**	**3,505**	**5,717**	**1,686**	**202**	**1,165**
Upregulated	**1,495**	**1,805**	**2,882**	**1,436**	**51**	**971**
Downregulated	**1,117**	**1,700**	**2,835**	**250**	**151**	**194**

PI = Periinfarct, pMCAO = permanent middle cerebral artery occlusion.

*P*<0.05; logFC ≥ |1|.

**Figure 3:**
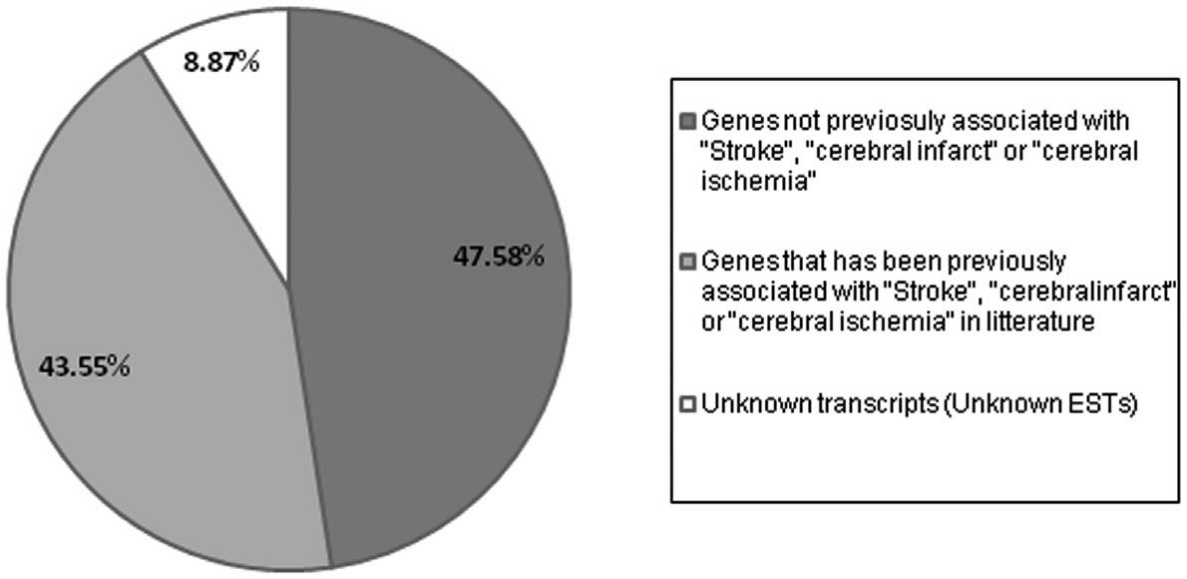
Microarray analysis leads to the identification of hundreds of genes not previously associated with stroke. Pie-chart showing the percentage of genes already associated with stroke or cerebral ischemia, the percentage of genes that were not associated, and the percentage of EST’s identified in this study. Percentages were calculated from a list of 300 genes selected by chance from the output of Venn diagrams and considering all the experimental conditions (24 h, 3 d, core and periinfarct). (EST: Expressed sequence tag).

To identify common regulatory responses, we clustered the genes into Venn diagrams according to their type of regulation (up/down), brain region and also time after ischemia induction (**Figure 2B**). The expression of more than 300 genes (both up- and down-regulated) changed specifically in the core of the infarct at 24 h compared with healthy animals, and more than 700 genes were altered in the PI area at the same time. After 3 d, more than 2,500 genes were regulated in the core of the lesion but only 13 genes were found to be specific to the PI. Thus, different patterns were observed. A total of 120 genes that were elevated in the PI area at 24 h remained upregulated after 3 d, whereas another subset of 324 genes upregulated at 24 h (and common to both the core and PI areas) was not detected after 3 d. Indeed, a total of 539 new genes found to be common to both core and PI at 3 d had not been elevated before. A total of 820 genes (out of 7341 regulated transcripts) were common to all times and areas. In order to compare our data with previous findings, 300 genes (randomly selected among all the conditions) were manually curated in search of a relation to “cerebral ischemia”, “cerebral infarct” or “stroke” (http://www.ncbi.nlm.nih.gov/pubmed/). Forty-seven percent of the regulated genes were found to be associated with one or more of the mentioned terms whereas almost half of the genes (43%) did not seem to have been previously mentioned in any experimental or clinical stroke study (**Figure 3**). These results indicate that our study not only validates previous findings but also extends our knowledge of ischemia-induced gene regulation.

### Biological Functions Regulated in Core and Periinfarct after Cerebral Infarct

**Figure 4:**
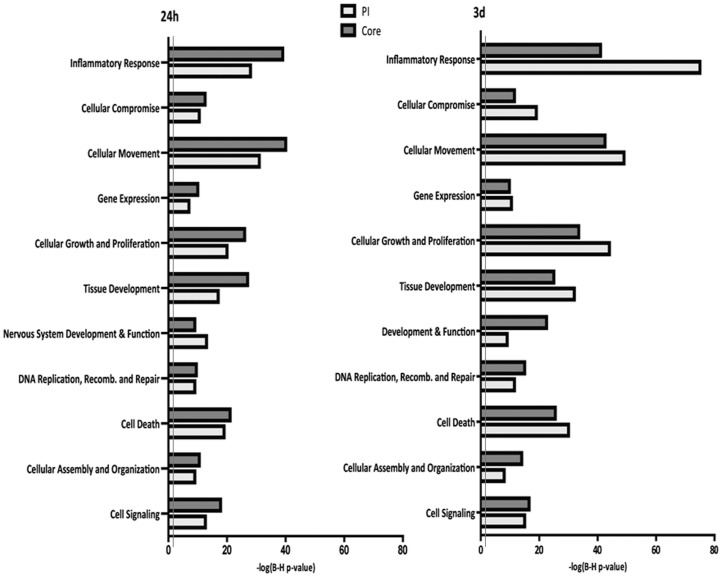
Biological functions that were significantly regulated in both core and periinfarct following focal cerebral ischemia. Biological functions calculated using Ingenuity pathway analysis (IPA) showed that all functions were significantly regulated in both core and periinfarct (PI) following pMCAO in both the 24 hour and 3 day groups (*P*<0.05; B – H Multiple testing p-value correction was selected).

To identify biological functions that were significantly altered in brain areas subjected to infarct in comparison with healthy animals, all the regulated genes were clustered according to several functional categories using Ingenuity Pathway Analysis software (www.ingenuity.com), (**Figure 4**).

24 h and 3 d after permanent ischemia the most altered IPA biofunctions were cellular movement and inflammatory response. Growth and proliferation, cell death, cell signaling and tissue development-associated genes were highly up- and down- regulated after cerebral infarct in core and PI areas.

#### Inflammatory response

The expression of multiple cytokines and their receptors was dysregulated in the microarrays, including *Interleukin (IL)-1Beta*, *IL-18*, *IL-33* and also the receptors *IL-4RA*, *IL-6RA*, *IL-7R*, *IL13RA1* and *IL17RE* at both 24 h and 3 d (**Table 2**). Additional regulated genes included *HLA-DRA* and *HLA-DRB* as well as genes related to cell division and expansion of immune cells. Some of the genes were only regulated at one or the other time-point; an example is *IL-33*, which was significantly altered at 24 h after cerebral infarct in the PI area, but not at 3 d.

**Table 2:** Regulation of inflammatory response and cell movement-associated genes in the core and perriinfarct after permanent focal cerebral ischemia.

Symbol	Gene Name	24 h		3 d	
		Core	Periinfarct	Core	Periinfarct
**Inflammatory Reponse**					
*TLR2*	Toll-Like Receptor 2	2.59 (<10^−4^)	2.36 (<0.01)	8.34 (<10^−10^)	4.00 (<10^−4^)
*IL1RL1*	Interleukin 1 receptor-like 1	13.8 (<10^−4^)	15.5 (<10^−5^)	57.7 (<10^−8^)	12.2 (<10^−4^)
*IL1B*	Interleukin-1 beta	21.9 (<10^−7^)	13.3 (<10^−6^)	13.5 (<10^−6^)	3.89 (<0.05)
*IL4RA*	Interleukin-4 receptor, alpha	4.17 (<10^−5^)	4.06 (<10^−5^)	5.24 (<10^−7^)	2.53 (<0.01)
*IL6RA*	Interleukin-6 receptor, alpha	2.87 (<10^−6^)	3,43 (<10^−8^)	3.92 (<10^−9^)	2,53 (<10^−5^)
*IL7R*	Interleukin-7 receptor	2.42 (<0.05)	2.13 (<0.05)	5.24 (<10^−5^)	3.23 (<0.01)
*IL13RA1*	Interleukin 13 receptor, alpha 1	2.81 (<0.01)	2.69 (<0.01)	5.28 (<10^−6^)	2.48 (<0.05)
*IL17RE*	interleukin 17 receptor E	**–**	4.63 (<10^−6^)	**–**	**–**
*IL18*	Interleukin-18	2.11 (<0.05)	2.73 (<10^−5^)	8.05 (<10^−11^)	3.53 (<10^−6^)
*IL33*	Interleukin-33	**–**	0.50 (<10^−5^)	**–**	**–**
**Cellular Movement**					
*SEMA3A*	Semaphorin-3A	**–**	**–**	0.34 (<10^−5^)	**–**
*SEMA4F*	Semaphorin-4F	**–**	0.46 (<10^−5^)	**–**	**–**
*NOS3*	Nitric oxide synthase 3	2.62 (<10^−4^)	2.49 (<10^−4^)	**–**	**–**
*GUCY1A3*	Guanylate cyclase soluble subunit alpha 3	0.29 (<10^−6^)	0.42 (<10^−4^)	0.40 (<10^−4^)	**–**
*ADM*	Adrenomedullin	4.29 (<10^−5^)	3.92 (<10^−5^)	3.97 (<10^−6^)	2.06 (<0.05)
*HAS1*	Hyaluronan synthase 1	144.01 (<10^−9^)	145.2 (<10^−9^)	14.52 (<10^−5^)	4.92 (<0.05)
*CXCR4*	C-X-C chemokine receptor type 4	**–**	**–**	9.51 (<10^−8^)	3.25 (<0.01)
*COL4A1*	Collagen, type IV, alpha 1	3.73 (<10^−4^)	4.11 (<10^−5^)	5.43 (<10^−6^)	2.62 (<0.01)
*FN1*	Fibronectin	2.36 (<10^−4^)	3.27 (<10^−5^)	8,18 (<10^−9^)	3.36 (<10^−4^)
*ICAM1*	Intercellular Adhesion Molecule 1	5.73 (<10^−7^)	5.54 (<10^−7^)	5.31 (<10^−7^)	2.64 (<0.01)
*PAK1*	Serine/threonine-protein kinase PAK 1	**–**	0.45 (<10^−5^)	0.44 (<10^−5^)	**–**
*S100A9*	S100A9	20.4 (<10^−5^)	15.1 (<10^−4^)	29.7 (<10^−6^)	8.81 (<0.01)
*SLIT2*	calgranulin-B	**–**	0.49 (<10^−5^)	**–**	**–**
*STAT3*	Signal transducer and activator of transcription 3	3.97 (<10^−8^)	3.29 (<10^−6^)	2.66 (<10^−4^)	**–**
*NRCAM*	Neuronal cell adhesion molecule	–	0.44 (<10^−5^)	0.41 (<10^−6^)	**–**

Expression is given by fold change in value compared with healthy controls. Adjusted p-value (Adj*P*) is given in parenthesis.

#### Cellular movement

Specific changes related to tissue remodeling and cell migration observed in the core and the PI areas included *SEMA3A* and *SEMA4F* (downregulated 24 h after stroke in the PI area) (**Table 2**). Also, the *NOS3* gene was upregulated 24 h after cerebral infarct in both areas but not after 3 d. *CXCR4* was upregulated 3 d after stroke in both the core and PI area. *ICAM1*, *ADM*, *FN1* and *S100A9* were upregulated in all samples at both times.

#### Gene expression

The expression of key transcription factors for cell development and proliferation was altered (**Table 3**). *HIF1A* mRNA levels were augmented at 24 h after stroke as were levels of *REST*, a repressor of neuronal differentiation that was upregulated in the core and PI area at 24 h and 3 d after cerebral infarct. The same was observed with *MYC* and *FAS*. *SMAD1* (related to *BMP* signaling) and *HEY1* (related to Notch signaling) were significantly altered only in the 24 h samples.

**Table 3:** Regulation of gene expression and tissue development-associated genes in the core and periinfarct after permanent focal cerebral ischemia.

Symbol	Gene Name	24 h		3 d	
		Core	Periinfarct	Core	Periinfarct
**Gene Expression**					
*REST*	RE1-silencing transcription factor	2.33 (<0.01)	2.97 (<0.01)	3,81 (<10^−6^)	2.16 (<0.05)
*SMAD1*	SMAD family member 1	2.35 (<10^−9^)	2.69 (<10^−11^)	**–**	**–**
*STAT4*	Signal transducer and activator of transcription 4	0,49 (<0.01)	**–**	0.35 (<10^−6^)	–
*FAS*	TNF receptor superfamily, member 6	3.73 (<10^−5^)	4.08 (<10^−5^)	7.16 (<10^−8^)	4.32 (<10^−5^)
*MYC*	Myc proto-oncogene protein	7.62 (<10^−9^)	6.87 (<10^−8^)	6.15 (<10^−9^)	3.01 (<10^−4^)
*HIF1A*	Hypoxia-inducible factor 1, alpha	2.11 (<10^−8^)	**–**	**–**	**–**
*ARNT2*	Aryl hydrocarbon receptor nuclear translocator 2	**–**	**–**	**–**	**–**
**Tissue Development**					
*NOTCH3*	Neurogenic locus notch homolog protein 3	**–**	2.14 (<0.05)	2,45 (<0.05)	**–**
*NOCTH4*	Neurogenic locus notch homolog protein 4	**–**	2.03 (<10^−4^)	**–**	**–**
*NOG*	Noggin	**–**	**–**	0,42 (<10^−6^)	**–**
*SOX7*	Sox-7 transcription factor	6.77 (<10^−7^)	5.03 (<10^−6^)	3.97 (<10^−6^)	2.20 (<0.05)
*HES1*	Transcription factor HES-1	**–**	**–**	**–**	**–**
*HES2*	Transcription factor HES-2	**–**	3.14 (<0.05)	**–**	**–**
*HES5*	Transcription factor HES-5	0.18 (<10^−6^)	0.16 (<10^−6^)	0.41 (<0.01)	**–**
*NEUROD1*	Neurogenic differentiation 1	0.40 (<10^−5^)	**–**	0.36 (<10^−6^)	**–**
*MMP2*	Matrix metalloproteinase-2	**–**	**–**	5.82 (<10^−10^)	3.81 (<10^−6^)
*MMP7*	Matrix metalloproteinase-7	–	**–**	81,0 (<10^−7^)	13,0 (<0.01)
*MMP9*	Matrix metalloproteinase-9	5.94 (<10^−6^)	4.03 (<10^−5^)	3.01 (<10^−4^)	2.31 (<0.05)
*MMP12*	Matrix metalloproteinase-12	24,8 (<10^−5^)	27,7 (<10^−5^)	125 (<10^−8^)	22,3 (<10^−4^)

Expression is given by fold change in value compared with healthy controls. Adjusted p-value (Adj*P*) is given in parenthesis.

#### Tissue development

Several matrix-methaloproteinases such as *MMP2*, *MMP7*, *MMP9* and *MMP12,* were up-regulated after cerebral infarct. *MMP2* and *MMP7* were only regulated after 3 d (**Table 3**). *NEUROD1* was only down-regulated in the core of the lesion. *NOG* gene (*Noggin*), was downregulated in the PI area at 3 d and *HES5*, involved in neurogenesis, was downregulated in both the core and PI areas at 24 h but only in the core of the lesion at 3 d after cerebral infarct.

#### Cellular growth and proliferation

Many genes related to cell division were also regulated (**Table 4**). *NRP1* was observed to be elevated only in the core 3 d after stroke and *NDN* gene was down-regulated in the PI area at 24 h. Except for *VEGFC* at 24 h after cerebral infarct, which was lower, the other *VEGF* genes (A and B) were not significantly altered at either time-point. In general, many regulators of the cell cycle showed elevated expression levels, including *CDC2*, *CDK2, CCNA2*, *CCND1*, *S100A6* and *RHOH*. The *MAP2* gene was not regulated although there was a tendency toward lower levels in the core at 3 d. The mRNA levels of *BAI1* were decreased at both 24 h and 3 d after stroke in the core but only at 24 h in the PI area.

**Table 4:** Regulation of cell growth and proliferation-associated genes in the core and periinfarct after permanent focal cerebral ischemia.

Symbol	Gene Name	24 h		3 d	
		Core	Periinfarct	Core	Periinfarct
**Cell Growth and Proliferation**					
*CDC2*	Cyclin-dependent kinase 1	5.73 (<0.01)	4.17 (<0.05)	64,5 (<10^−9^)	20,1 (<10^−5^)
*CDK2*	Cyclin-dependent kinase 2	**–**	**–**	2,75 (<0.05)	2.03 (<0.01)
*MAP2*	Microtubule-associated protein 2	**–**	**–**	**–**	**–**
*CDK5R1*	Cyclin-dependent kinase 5,regulatory subunit 1	**–**	**–**	0.46 (<0.01)	**–**
*CCNA2*	Cyclin A2	3.95 (0.01)	3.20 (<0.01)	33,9 (<10^−10^)	10,3 (10^−6^)
*CCND1*	Cyclin D1	2.48 (<10^−4^)	2,00 (<0.01)	6.15 (<10^−9^)	2.79 (10^−4^)
*NRP1*	Neuropilin 1	–	–	2.07 (<10^−7^)	–
*BAI1*	Brain-specific angiogenesis inhibitor 1	0.49 (<10^−4^)	0.46 (<0.05)	0.37 (<0.01)	–
*NDN*	Necdin	–	0.40 (<10^−5^)	–	–
*S100A6*	S100A6 gene	3.58 (<10^−4^)	6.28 (<10^−6^)	11,0 (<10^−9^)	5.39 (<10^−5^)
*INHBA*	Inhibin, beta A	6.77 (<10^−4^)	**–**	**–**	**–**
*RUNX3*	Runt-related transcription factor 3	**–**	**–**	**–**	**–**
*MAP4K1*	Mitogen-activated protein kinase 1	**–**	**–**	4.69 (<10^−7^)	2.03 (<0.05)
*RHOH*	Ras homolog gene family, member H	2.08 (<0.05)	2.11 (<0.05)	6.54 (<10^−7^)	2.95 (<0.01)
*VEGFA*	Vascular endothelial growth factor A	**–**	**–**	**–**	**–**
*VEGFB*	Vascular endothelial growth factor B	–	–	–	–
*VEGFC*	Vascular endothelial growth factor C	0.42 (<10^−8^)	–	–	–
*FGF13*	Fibroblast growth factor 13	0.47 (<10^−5^)	0.46 (<10^−5^)	0.33 (<10^−8^)	–
*GNAO1*	Guanine nucleotide binding protein	–	–	–	–

Expression is given by fold change in value compared with healthy controls. Adjusted p-value (Adj*P*) is given in parenthesis.

#### Nervous system development and function

The expression of many trophic factors as well as several neurogenesis- and oligodendrogenesis-related genes was altered (**Table 5**). The oligodendrocyte differentiation repressor *LINGO1* was downregulated in the PI area at 24 h and remained at lower expression values in the core of the lesion after 3 d of ischemia. On the other hand, 24 h and 3 d after occlusion, *RTN4* (*Nogo-A*) and its receptor were downregulated in both the core and PI areas while *OLIG2* was downregulated in the core only at 24 h. *NRCAM*, *SYN1*, *GRIN1*, and *ROBO2* were downregulated in the PI area but not core at 24 h and in the core but not PI at 3 d. Most of the trophic factors showed augmented levels after stroke. *NTF4*, *HB-EGF* and *TGFB2* were already augmented at 24 h in both core and PI areas whereas at 3 d *TGFB1*, *GFAP* and *FGF2* expression levels had also risen in both areas. *GRN* (*Granulin*) gene expression was augmented after 3 d. *BMP4* levels were diminished at 24 h and *BMP7* levels were augmented in the core of the lesion at 3 d. Some genes like *BDNF* presented a differential response, since their levels were augmented at 24 h but had decreased at 3 d of ischemia.

**Table 5:** Regulation of nervous system development and function-associated genes in the core and periinfarct after permanent focal cerebral ischemia.

Symbol	Gene Name	24 h		3 d	
		Core	Periinfarct	Core	Periinfarct
**Nervous System Dev. & Function**					
*SYN1*	Synapsin I	–	0.40 (<10^−5^)	0.42 (<10^−5^)	–
*TF*	Transferrin	2.16 (<0.01)	**–**	2.68 (<10^−5^)	2.11 (<0.01)
*LINGO1*	Leucine rich repeat and Ig domain containing 1	–	0.44 (<10^−4^)	0.33 (<10^−5^)	–
*RTN4 (NOGO)*	Reticulon-4	0.44 (<0.01)	0.34 (<10^−4^)	0.35 (<10^−4^)	0.49 (<0.05)
*RTN4R*	Reticulon-4 receptor	0.50 (<10^−4^)	0,.40 (<10^−5^)	0.39 (<10^−6^)	–
*BMP4*	Bone morphogenetic protein-4	0.40 (<10^−5^)	0.43 (<10^−4^)	–	–
*BMP7*	Bone morphogenetic protein-7	–	–	2.39 (<10^−5^)	–
*GRN*	Granulin	–	–	10.3 (<10^−11^)	4.76 (<10^−6^)
*GRIN1*	Glutamate [NMDA] receptor subunit zeta-1	–	0.49 (<10^−5^)	0.41 (<10^−7^)	–
*ROBO2*	Roundabout, axon guidance receptor, homolog 2	–	0.49 (<10^−4^)	0.46 (<10^−5^)	–
*GFAP*	Glial fibrillary acidic protein	7.52 (<10^−6^)	10.1 (<10^−7^)	11.5 (<10^−8^)	9.19 (<10^−6^)
*FGF2*	Fibroblast growth factor 2	3.65 (<10^−8^)	3.94 (<10^−8^)	–	2.16 (<10^−4^)
*HB-EGF*	Heparin-binding EGF-like growth factor	4.23 (<10^−5^)	3.41 (<10^−4^)	–	–
*TGFB1*	Transforming growth factor, beta 1	3.61 (<10^−4^)	2.71 (<0.01)	8.81 (<10^−8^)	3.61 (<10^−4^)
*TGFB2*	Transforming growth factor, beta 2	3.05 (<10^−10^)	–	–	–
*BDNF*	Brain-derived neurotrophic factor	2.66 (<0.05)	–	0.27 (<0.01)	–
*NTF4*	Neurotrophin 4	2.27 (<0.01)	3.81(<10^−6^)	–	–
*ARTN*	Artemin	2.81 (<0.01)	**–**	–	–
*YWHAH*	14-3-3 protein eta	–	0.47 (<10^−4^)	–	–
*PTN (NEGF1)*	Pleiotropin	–	**–**	–	–
*NRCAM*	Neuronal cell adhesion molecule	–	0.44 (<10^−5^)	0.41 (<10^−6^)	–
*DCX*	Doublecortin	–	–	0.50 (<10^−4^)	–
*NUMB*	Protein numb homolog	–	–	–	–
*DPYSL3*	DPY-related protein 3	–	–	2.14 (<10^−4^)	–

Expression is given by fold change in value compared with healthy controls. Adjusted p-value (Adj*P*) is given in parenthesis.

#### DNA replication, recombination and repair

The stress response following stroke induced several replication-associated genes such as *CDT1* and *E2F3* (**Table 6**). At both 24 h and 3 d after cerebral infarct the expression of repair and recombination genes such as *EXO1* was higher. Levels of ADA were also increased at both time points.

**Table 6:** Regulation of DNA replication, recombination & repair and also cell death-associated genes in the core and periinfarct after permanent focal cerebral ischemia.

Symbol	Gene Name	24 h		3 d	
		Core	Periinfarct	Core	Periinfarct
**DNA Replication Recomb. & Repair**					
*ADA*	Adenosine deaminase	–	2.64 (<0.01)	6.19 (<10^−7^)	3.12 (<0.01)
*RPA1*	Replication protein A1	–	0.47 (<0.01)	–	–
*CDT1*	DNA replication factor CDT1	4.76 (<10^−4^)	3.58 (<0.01)	11.6 (<10^−7^)	4.00 (<0.01)
*E2F3*	Transcription factor E2F3	–	2.01 (<10^−4^)	–	–
*EXO1*	Exonuclease 1	3.53 (<10^−4^)	2.99 (<10^−4^)	13.1 (<10^−10^)	4.44 (<10^−5^)
**Cell Death**					
*CASP3*	Caspase 3	2.43 (<10^−6^)	–	–	–
*CASP9*	Caspase 9	2.13 (<0.05)	0.32 (<10^−4^)	0.43 (<0.01)	–
*BCL2*	B-cell lymphoma 2	–	0.49 (<0.01)	–	–
*AIFM3*	Apoptosis-inducing factor, mitochondrion-associated 3	0.32 (<10^6^)	0.34 (<10^5^)	0.30 (<10^7^)	–
*BID*	BH3 interacting-domain death agonist	–	–	–	–
*CASP8*	Caspase 8	–	–	4.23 (<10^−4^)	–
*BIRC5*	Survivin	–	–	13.1 (<10^−10^)	4.17 (<10^−4^)
*ADORA1*	Adenosine A1 receptor	–	0.47 (<0.01)	–	–
*HSP27 (HSP18)*	Heat shock protein 27	43.1 (<10^−9^)	48.8 (<10^−10^)	15.6 (<10^−7^)	9.78 (<10^−5^)
*HSPA1A (HSP72)*	Heat shock 70 kDa protein 1	52.0 (<10^−12^)	81.6 (<10^−14^)	2.56 (<0.01)	**–**
*HSPA1B*	Heat shock 70 kDa protein 1B	73.5 (<10^−16^)	129 (<10^−18^)	2.66 (<10^−4^)	**–**
*HSPA2*	Heat shock-related 70 kDa protein 2	3.09 (<10^−8^)	3.03 (<10^−7^)	**–**	**–**
*HSPA8 (HSC70)*	Heat shock 70 kDa protein 8	–	–	–	–
*HSPA9 (GPR75)*	Heat shock 70 kDa protein 9	–	2.25 (<10^−7^)	–	–

Expression is given by fold change in value compared with healthy controls. Adjusted p-value (Adj*P*) is given in parenthesis.

#### Cell death

Expression values of genes belonging to both the extrinsic and intrinsic pathways of apoptosis changed. *CASP3* was augmented 24 h after stroke in the core of the lesion, whereas *CASP9* and *AIFM3* levels were decreased after cerebral infarct in both core and PI areas, but these differences with normal levels were not significant in the PI area 3 d after the lesion (**Table 6**). Levels of *BCL2* and *ADORA1* were decreased at 24 h in the PI area although *BID* mRNA levels were not significantly altered. *CASP8* and *BIRC5* genes were elevated after 3 d. Looking at chaperonins, *HSP27*, *HSPA1A*, *HSPA1B* and *HSPA2* were always increased in both areas at both time-points, although the increase in the PI area at 3 d did not quite reach significance.

#### Cellular assembly and organization

*RHOA* expression did not change at either time-point, whereas the levels of *FEZ1* and *TNR* diminished in the PI area at 24 h (**Table 7**). *SMC4*, *NUF2* and *ECT2* were augmented in the core and PI areas at 3 d after stroke.

**Table 7:** Regulation of cellular assembly & organization and cell signaling-associated genes in the core and periinfarct after permanent focal cerebral ischemia.

Symbol	Gene Name	24 h		3 d	
		Core	Periinfarct	Core	Periinfarct
**Cellular Assembly & Organization**					
*RHOA*	Ras homolog gene family, member A	–	–	**–**	**–**
*SMC4*	Structural maintenance of chromosomes 4	**–**	**–**	3.83 (<10^−9^)	2.02 (<10^−4^)
*NUF2*	Kinetochore protein Nuf2	**–**	**–**	19.7 (<10^−9^)	7.36 (<10^−5^)
*ECT2*	ECT2 gene	3.16 (<0.05)	**–**	32.0 (<10^−10^)	10.6 (<10^−5^)
*FEZ1*	Fasciculation and elongation protein zeta-1	**–**	**–**	0.45 (<0.01)	**–**
*TNR*	Tenascin-R	–	–	0.47 (<10^−7^)	–
**Cell Signaling**					
*RFTN1*	Raftlin, lipid raft linker 1	2.12 (<0.01)	2.45 (<10^−4^)	7.06 (<10^−9^)	2.29 (<0.01)
*NDN*	Necdin	–	0.40 (<10^−5^)	–	–
*CCL5*	Chemokine (C-C motif) ligand 5	–	2.39 (<0.05)	2.36 (<0.01)	–
*CXCL1*	Chemokine (C-X-C motif) ligand 1	27.1 (<0.01)	238 (<10^−6^)	82.7 (<10^−5^)	27.7 (<0.01)
*CXCL10*	Chemokine (C-X-C motif) ligand 10	7.36 (<10^−4^)	9.38 (<10^−4^)	6.27 (<10^−4^)	9.32 (<10^−4^)
*CXCL11*	Chemokine (C-X-C motif) ligand 11	11.5 (<10^−4^)	22.8 (<10^−5^)	25.3 (<10^−6^)	12.8 (<10^−4^)
*CXCL12 (T1)*	Chemokine (C-X-C motif) ligand 12 (transcript 1)	–	–	–	–
*CXCL12 (T3)*	Chemokine (C-X-C motif) ligand 12 (transcript 3)	0.28 (<10^−9^)	0.36 (<10^−7^)	–	–
*CXCL13*	Chemokine (C-X-C motif) ligand 13	4.75 (<0.05)	3.48 (<0.05)	10.9 (<10^−5^)	5.89 (<0.05)
*CCL2*	Chemokine (C-C motif) ligand 2	576 (<10^−8^)	560 (<10^−8^)	388 (<10^−8^)	69.1 (<10^−5^)
*CCL4*	Chemokine (C-C motif) ligand 4	15.8 (<10^−7^)	14.0 (<10^−7^)	7.94 (<10^−6^)	2.88 (<0.05)
*CCL5*	Chemokine (C-C motif) ligand 5	–	2.39 (<0.05)	2.36 (<0.01)	–
*CCL20*	Chemokine (C-C motif) ligand 20	46.2 (<10^−5^)	90,5 (<10^−6^)	4.86 (<0.05)	–
*MyD88*	Myeloid differentiation primary response gene 88	2.27 (<0.01)	2.00 (<0.05)	2.71 (<10^−4^)	–
*ANGPT2*	Angiopoietin-2	3.41 (<0.05)	2.92 (<0.05)	2.36 (<0.05)	–
*CALCB*	Calcitonin-related polypeptide beta	18.4 (<10^−8^)	12.2 (<10^−6^)	61.8(<10^−11^)	11.8 (<10^−6^)

Expression is given by fold change in value compared with healthy controls. Adjusted p-value (Adj*P*) is given in parenthesis.

#### Cell signaling

Multiple genes related to cell signaling changed during the response to ischemia, and many of them showed increased RNA levels (e.g. *CXCL1*, *CXCL10*, *CXCL11* and *CXCL13*). At 24 h *CXCL12* levels were decreased in transcript 3 (**Table 7**) but there were no changes in this transcript at 3 d and none in transcript 1 at either of the studied time points. *CCL2*, *CCL4*, *CCL5*, *CCL20* and *MyD88* showed augmented levels and *ANGPT2* had elevated core levels both 24 h and 3 d after ischemia but not in PI at 3 d.

### Comparison between Core and PI Gene Expression

**Figure 5:**
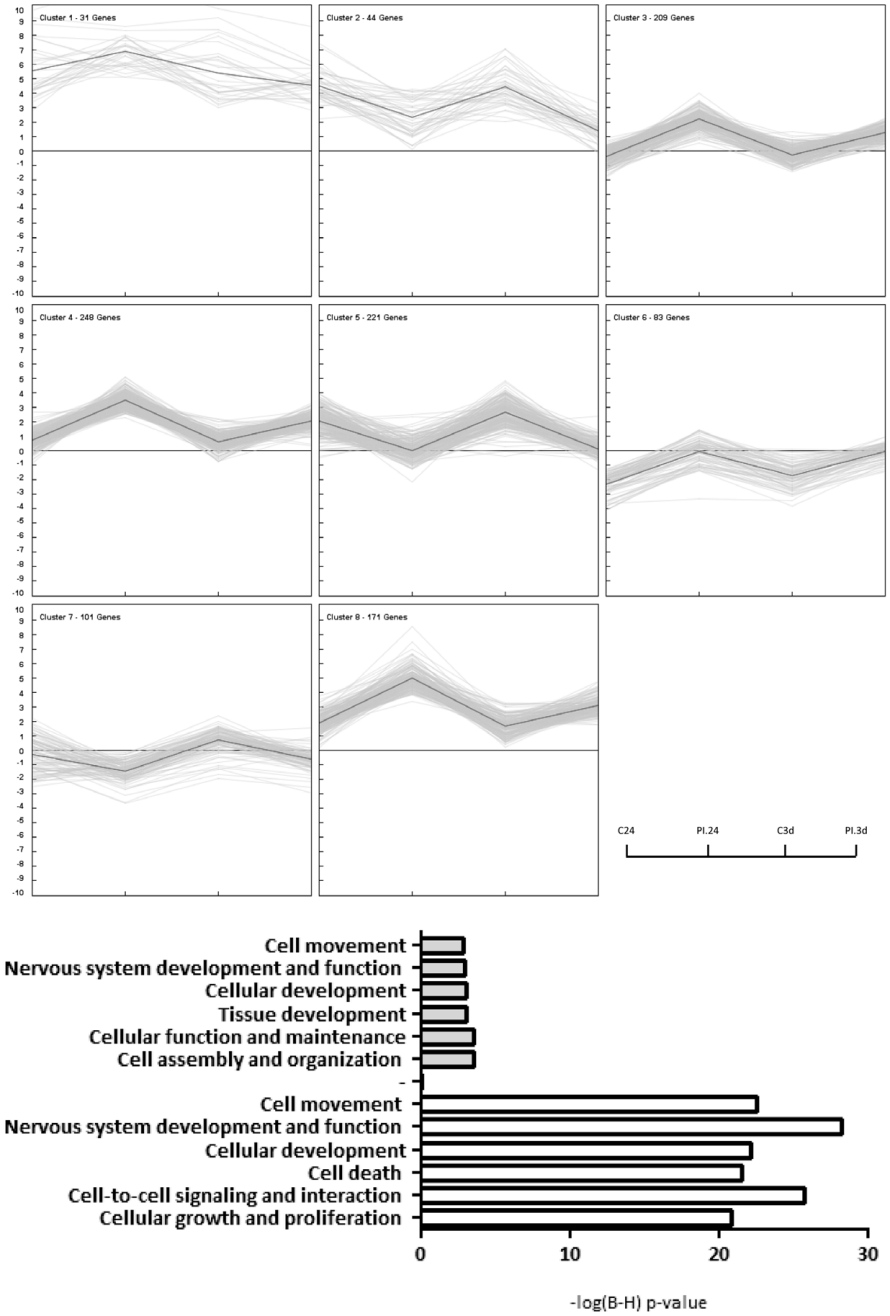
Differential gene regulation of core and periinfarct areas following pMCAO. a) k-Means clustering showing 8 different gene expression patterns. From left to right: Core at 24 h, Periinfarct at 24 h, Core at 3 d and Periinfarct area at 3 d; b) most significantly altered IPA biofunctions when comparing both areas at 24 h or 3 d of ischemia.

To go a step further in our analysis of expression level, the differences between the core and PI were statistically analyzed at 24 h and 3 d (**Table 1**). The number of differentially expressed genes increased from 202 at 24 h to 1,165 at 3 days. K-mean clustering revealed tendencies and common patterns for some of the genes and IPA functional analysis identified relationships between the genes whose expression levels had changed (**Figure 5**).

The most upregulated genes in the core at 24 h (Fold-Changes are given in parenthesis) were: *OPRK1* (4.73); *CBLN1* (4.67); *SLC6A5* (4.12); *CCL21* (3.23); *ATOH7* (3.16); *HAS2* (3.075); *CRABP1* (2.81); *HS3ST2* (2.806); *INHBA* (2.74); and *GIPR* (2.71). The most downregulated genes were *CCL11* (0.122); *SLC22A1* (0.153); *SLC6A20* (0.157); *DLX3* (0.157); *SFPQ* (0.170); *LNX2* (0.194); *DLK1* (0.197); *Cyp2b13/Cyp2b9* (0.208); *CLDN19* (0.222) and *ALDH1A2* (0.222). The most significant IPA-biofunctions [−log(B–H) p-value) is given] were cell assembly and organization (3.55), cellular function and maintenance (3.54), cellular development (3.05), tissue development (3.01), nervous system development and function (2.90) and, last, cell movement (2.87).

At 3 d, the most upregulated genes in the core (Fold Changes are given in parenthesis) were: *CD8A* (14.52); *FABP4* (14.82); *CLEC2A* (14.62); *HK3* (9.81); *PLAU* (7.96); *SPP1* (7.77); *CST7* (7.37); *SCIN* (7.35); *TREM1* (7.29) and *ATP6V0D2* (7.19). The most downregulated were: *ARR3* (0.236); *SLC12A1* (0.295); *CAMK2A* (0.318); *FRAS1* (0.335); *PRKG2* (0.345); *UNC45B* (0.347); *TNNC2* (0.350]; *SCGN* [logFC (0.317); *FCHO1* (0.354) and *WDR26* (0.37) and the most significant IPA-biofunctions [−log(B–H) p-value) is given] were nervous system development and function (28.3), cell-to-cell signaling and interaction (25.8), cellular movement (22.55), cellular development (22.1), cell death (21.5), and cellular growth and proliferation (20.9). There were differences in the levels of expression for many genes and also for several functional categories related to nervous system development and function between the core and PI.

### Validation of Microarray Data by qRT-PCR

**Figure 6:**
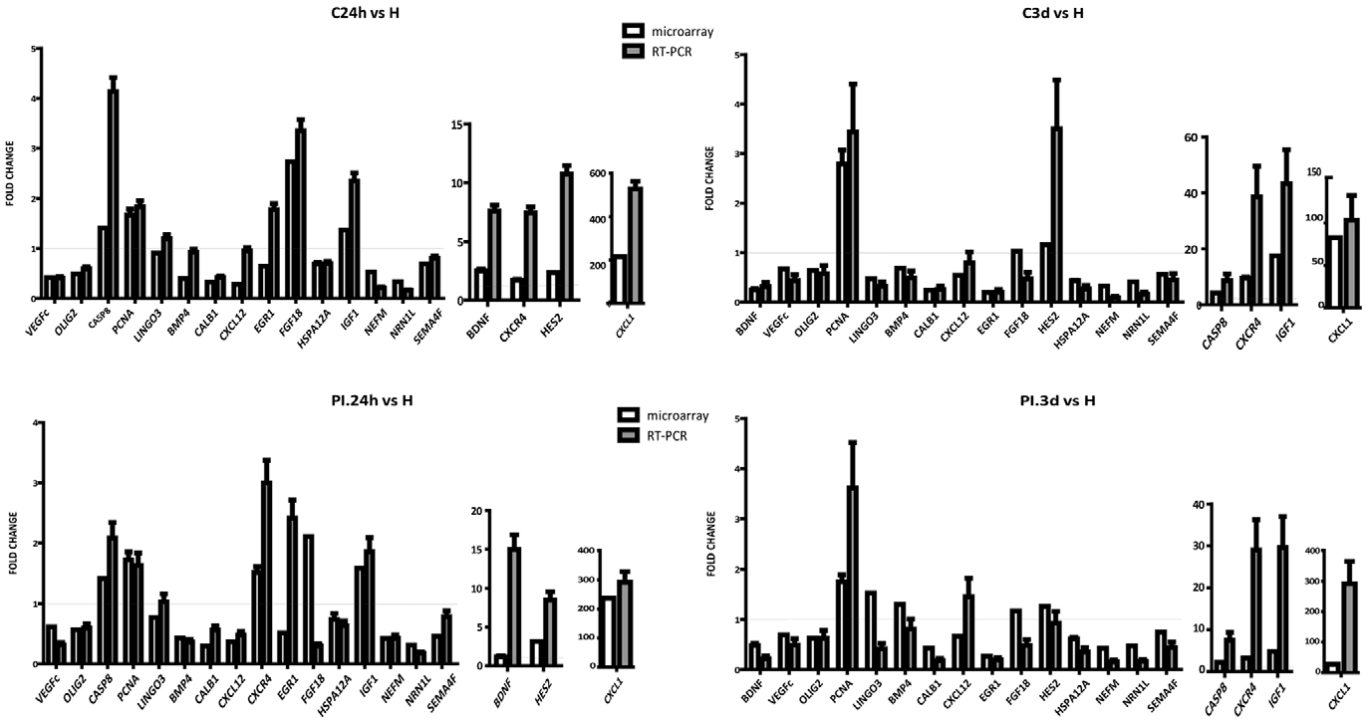
Validation of data from microarray analysis. The levels of 19 representative genes were assessed by qRT-PCR in a new set of 12 animals. Values were normalized to GAPDH, PGK1 and HMBS control genes. Data represent the means ± SD of three independent experiments performed in triplicate. Differences with healthy animals were arbitrarily set to 1. All changes in gene expression were significant (*P*<0.01). Red line indicates FC = 1.

In order to validate our results, a total of 22 genes (19 regulated genes plus 3 housekeeping genes) were analyzed by Real-Time (RT)-PCR (**Figure 6**). In general, the PCR findings and the microarray data showed good agreement regarding the magnitude of change of the logFC values.

## Discussion

To the best of our knowledge this is the most detailed study of gene expression following permanent focal cerebral infarct available to date. Thousands of transcripts were found to undergo expression regulation (p>2) at 24 h and 3 d following pMCAO in the ischemic core and surrounding periinfarct cortex.

The real differences between humans and animal models made it difficult to select specific time-points for our study that could also reflect the human situation. The initial analysis time of 24 h was selected because it has been reported that infarct size would be well stabilized in the pMCAO model by then and repair responses would probably be most detectable [10]. The second analytic time-point was 3 d since it would be difficult to distinguish a functional penumbra (compromised periinfarct) at more than 3 to 5 days after pMCAO in the rat. Furthermore, after this time-point what would probably be found would be late neuronal (and non-neuronal) cell death instead of pannecrosis from the infarct itself. The study examined a large number of individuals (60) so as to guarantee the validity of the results. Samples were pooled and hybridized to a feasible number of arrays. Marker candidates were verified using RT-PCR in an independent set of rats. While the use of pooled samples can help understand the processes undelying the events following focal cerebral infarct [16], a more thorough study in single individuals must be performed to validate potential biomarkers.

### Gene Expression Profiles in Cerebral Infarct (Core and PI), Sham and Healthy Groups

A large number of genes were found to be differentially expressed after permanent cerebral infarct; some of these differences did not change with either the area studied or the time elapsed since infarct and probably reflects common universal responses to ischemic stress as well as common repair mechanisms. Focused on this time-points, the study revealed different patterns of gene regulation at 24 h (acute phase) and at 3 d (delayed response).

Globally, we observed two independent time patterns in the core and PI areas. The number of regulated genes in the PI area decreased between 24 h and 3 d. In contrast, even though many changes were observed in the core at 24 h, there were even more at 3 d after the occlusion. Both areas presented strong regulation of gene expression at 24 h post-stroke, but the response was even more notable at the late stage (3 days). Previous reports suggested that the penumbra (in terms of volumetric analysis) might remain differentiated from the core from the first hours to as long as 3–5 days after ischemia under conditions of a permanent MCA occlusion [13]. Our results suggest gene expression is quite different at distinct pivotal time-points in the pMCAO model.

### Inflammatory Response and Cellular Movement

We were interested in identifying the biofunctions that were associated with the highest number of affected genes. With the permanent cerebral infarct, the most altered IPA-biofunctions were those for cellular movement and inflammatory response. Common ischemia-regulated genes, including *IL-1B* and *IL-13RA1*, were also reported in previous microarray reports of transitory ischemia [17]. As expected, pro- and anti-inflammatory receptors such as *IL-4RA*, *IL-6RA*, *IL-7R* and *IL-17RE* were also altered. Most of the observed changes probably reflect lymphocyte recruitment to the damaged lesion site. In our study the upregulation of *TLR2* was particularly marked at 3 d of permanent ischemia. It has been reported that activation of the toll-like receptor (TLR) signaling pathway exacerbates ischemic brain damage and, in fact, TLR ligand administration has been shown to promote a certain tolerance by reprogramming TLR function [18]. Indeed *TLR2* signaling suppression could also be a valuable approach to minimizing postischemic inflammation that might have clinical interest. [19].

The expression levels of a large number of transcripts related to cellular movement were also altered. In our data *CXCR4* was upregulated at 3 d after permanent occlusion in both the core and PI area and this result was confirmed by qRT-PCR. Previous reports on permanent ischemia in mice describe *CXCR4* fluorescent staining in white matter as well as PI areas at 3 d and later [20]. CXCR4 upregulation might reflect migration of immune cells but also of neuroblasts and vasculature remodeling after damage. Indeed, pharmacological modulation of CXCR4 signaling has recently been used as a therapeutic approach [21].

Many methalloproteinases showed highly altered expression, including *MMP2* and *MMP7* at 3 d after cerebral infarct, as well as *MMP9* and *MMP12* in the acute (24 h) phase. All these changes suggest tissue matrix and cell-interaction remodeling from the first stages of a cerebral infarct. In a recent study of MMP inhibition after transitory ischemia, treatment with Ro 28-2653 was effective during the first two days but not at later times or for longer periods [22]. Consequently, MMP inhibition could be useful only at specific stages of remodeling.

### Cellular Growth & Proliferation and CNS Development

Multiple genes associated with neurovascular development were also affected. *LINGO1,* a repressor of oligodendrocyte development, was downregulated in the periinfarct area 24 h following stroke as was *RTN4 (NOGO-A)* and its receptor *RTN4R. LINGO1* was still significantly downregulated in the core after 3 days. These changes might reflect a decrease in the number of precursor and mature oligodendrocytes, as other authors have suggested may occur 48 hours after a transient cerebral infarct [23]. The decreased levels of both *LINGO1* and *NOGO-A* might initially seem to be due to a lower number of OPCs and also OLCs, but the *transferrin* gene (*TF*), which is only found in oligodendrocytes in the adult brain [24], was upregulated at both 24 h and 3 d after stroke. Although hard to interpret by only examining RNA levels, the reduction in *LINGO1* and *NOGO-A* might reflect an early activation of remyelination in immature OPCs.

The expression values of important genes for neurogenesis such as HES1, HES2, HES5, SOX2, SOX6 and SOX7 were also altered. *HES2* was upregulated in the PI area and this was confirmed by qRT-PCR. *HES5* was strongly downregulated and *SOX2* also showed a tendency to be decreased in the acute phase. Since inactivation of both *HES1* and *HES5* has been reported to promote neurogenesis [25], *HES1* could be a potential therapeutic target in ischemic conditions. A possible future strategy might be to check the levels of some *SOX6* and *HES5* regulators such as miR-219 or miR-338 and their therapeutic potential in stroke [26]. On the other hand, the *SOX7* gene, which has not previously been associated with stroke, was highly upregulated at both 24 h and 3 d after ischemia in the core and PI areas and recent data suggest it has a role in vascular development [27]. This would make it interesting to analyze the exact role of *SOX7* in post-stroke angiogenesis.

The expression of neuronal and synaptic markers such as *DCX (doublecortin)* and *SYN1 (Synapsin)* was diminished. *NUMB* gene expression was also decreased, though not significantly, and the *NEUROD1* gene was also downregulated. *SYP (Synaptophysin)* was also diminished in the core after 3 d. Most of these data seem to suggest acute inhibition of neurogenesis, but the *SYPL2* gene (*Synaptophysin-like-2*) and the neurogenesis marker *DPYSL3 (TUC-4)* were augmented in the core at 3 d after cerebral infarct, probably reflecting early brain repair responses after damage.

In transient models of ischemia there is a rapid upregulation of *VEGF* [28]. Nevertheless, the role of *VEGF* remains unclear. Its expression after ischemia increases permeability and therefore the risk of edema [29]. In our permanent ischemia model two of the *VEGF* genes (A and B) were not upregulated at 24 h or 3 d after permanent ischemia, although *VEGFC* was downregulated at 24 h but not at 3 d. This result was confirmed by PCR, and the biological relevance of this observation, if any, is still unknown. We detected increased levels of the transcription factor *HIF1A* during the acute phase at 24 h and also of some of its downstream targets like *ADM (Adrenomedullin)* or *ANGPT2 (Angiopoietin)*, in agreement with earlier reports [30].

Multiple trophic factors were also upregulated at 24 h including *NTF4, FGF2, TGFB1* and *BDNF*. All these observations might reflect acute repair responses that are activated to induce neurogenesis and oligodendrogenesis in the post-ischemic brain within 24 h. It has been suggested that brain repair responses start at the very beginning of the infarct [8]. Gene expression levels change at different stages. *BDNF* upregulation was observed in the acute phase but, surprisingly, it was downregulated at 3 d and this result was also confirmed by PCR, meriting further investigation. The upregulation of BDNF has been reported by other authors [30]. In a recent paper elevated levels of BDNF have been observed at 4 h and 24 h after stroke [31] but the putative role of *BDNF* as a peripheral marker in patients has been questioned as significant changes have not been observed in the first 4 days [32]. We wonder whether the negative result might somehow be related with the inversion observed here.

### Cell Death and Cell Signaling

The total number of cytokines and chemokines that are activated following ischemic stress is notable. It seems that stroke response involves an acute activation of inflammation followed by immunosuppression. We observed a rapid upregulation of multiple cytokines and chemokines after permanent ischemia including *CXCL1*, *CXCL10*, *CXCL11* and also *CCL2*, *CCL4*, *CCL5* and *CCL20.* All these changes may reflect signaling that allows lymphocytes and stem cells to home in on the infarcted site and it would be interesting to analyze their role as possible peripheral markers.

In contrast with what we expected due to previous reports describing SDF1 upregulation [33], we did not observe substantial *SDF1 (CXCL12)* upregulation in either the microarrays or the PCR after permanent ischemia. Indeed, we detected decreased *SDF1* levels in the core of the lesion and also in PI areas at 24 h. This tendency toward downregulation continued at 3 d. It is possible that an early response after permanent injury would decrease its levels with expression returning to normal and then upregulated values after 4 days of ischemia as has been shown in rats after permanent ischemia at 7 d [34]. Whatever the case, it is clear that strategies dedicated toward pharmacologically raising SDF1 levels have shown efficacy and are promising [21].

We also observed differences in cell death and heat shock protein (HSP) genes after permanent ischemia. There was a global upregulation of HSP’s including *HSP2, HSPA9, HSP27, HSPA1A (HSP72).* It has previously been reported that *HSP27* expression is augmented at 24 h following both mild and severe ischemia, mainly in astrocytes but also in neurons in different ischemic regions [35]. In addition to their relevance as possible targets for pharmacological therapy, we would note that they may be interesting potential markers in peripheral blood during the acute phase.

As could be expected due to the intrinsic stress caused by ischemia, the expression levels of several genes participating in cell death programs were changed. These results, however, should be carefully interpreted as both intrinsic and extrinsic apoptosis pathways are strongly regulated at the posttranscriptional level. Nevertheless, we observed specific changes at the RNA level for *BCL2*, *CASP8*, *CASP9* and *AIFM3*. We also detected upregulation of *Fas*, *FasL*, and TNF-related apoptosis-inducing ligands in agreement with previous reports [36].

### Last Comments

This is a comprehensive microarray study analyzing gene expression responses in different areas of the rat brain following permanent focal cerebral ischemia. We particularly examined different times at which natural repair responses are most likely to occur and detected multiple regulations of genes related to neuronal, oligodendrocyte and vascular development as well as cellular movement and tissue reorganization. The information provided here is of potential utility for both the identification of new markers and therapeutic targets as well as for the development of new clinical strategies for translational research in stroke.

## Materials and Methods

### Ethics Statement

All experiments were designed to minimize suffering in compliance with our medical school’s Ethical Committee for the Care and Use of Animals in Research (following EU directives 86/609/CEE and 2003/65/CE).

### Animals and Surgery

A total of 72 Sprague-Dawley rats weighting 250–320 gr. (Harlan Iberica S.L.) were used; 60 for microarray analysis and 12 additional animals for qRT-PCR validation. There were 5 groups: healthy unoperated rats (n = 12); ischemic rats at 24 h and 3 d survival (n = 12 respectively) and sham-operated animals at 24 h and 3 d survival (n = 12 respectively). To induce cerebral infarct, the middle cerebral artery was occluded with a permanent 9-0 suture in a 3VO model as previously described [37]. Briefly, animals were anesthetized before surgery with a solution of ketamine (25 mg/kg), diazepam (2 mg/kg), and atropine (0.1 mg/kg) at a volume of 2.5 ml/kg by intraperitoneal injection. Analgesia was provided with meloxicam 2 mg/kg by subcutaneous administration and a small craniotomy was made above the rhinal fissure over the branch of the right middle cerebral artery (MCA), which was permanently ligated just before its bifurcation between the frontal and parietal branches. Both common carotid arteries were then transiently occluded for 60 min. Complete interruption of blood flow was confirmed using an operating microscope and indirectly confirmed by the presence of an ischemic lesion in magnetic resonance imaging (MRI) and ADC maps of the brains. A thermistor probe was placed under the temporal muscle and over the cerebral artery region to measure brain temperature while glucose, blood gases and blood pressure were monitored (Monitor Omicron ALTEA RGB medical devices). Cranial and body temperature were maintained at 36.5±0.5°C during surgery and other physiological parameters (glycemia, blood gases and blood pressure) were maintained within 20% deviation from normal values An identical protocol was applied to Sham-operated rats, which were subjected to exactly the same surgery protocol but without occlusion. All rats were fed ad libitum in their home cages until sacrifice at 24 h or 3 days.

### Microarrays Assay, Data Pre-processing and Data Analysis

The 3VO model has been used before to study the metabolic evolution of core and periinfarct areas [15]. Following the same protocol we obtained highly reproducible cortical infarcts from which core and peri-infarct areas were dissected out from the brains for RNA isolation (**Figure 1**) using a commercial kit following the manufacturer’s instructions (Qiagen RNeasy kit, Hilden, Germany). Integrity and quantity were checked with the Agilent Bio-Analyzer QC. Ipsilateral and contralateral samples from healthy and sham-operated animals were also examined.

One-Color Microarray-Based Gene Expression Analysis Protocol (Agilent Technologies, Palo Alto, CA, USA) was used to amplify and label RNA. Samples were hybridized on Whole Rat Genome Microarrays 4×44 K (G4131F, Agilent Technologies). 1.65 micrograms of Cy3-labeled aRNA were hybridized for 17 hours at 65°C in an Agilent hybridization oven (G2545A, Agilent Technologies) set at 10 rpm in a final concentration of 1× GEx Hybridization Buffer HI-RPM (Agilent Technologies). Arrays were washed and dried using a centrifuge according to the manufacturer's instructions (One-Color Microarray-Based Gene Expression Analysis, Agilent Technologies). Arrays were scanned at 5 mm resolution on an Agilent DNA Microarray Scanner (G2565BA, Agilent Technologies) using the default settings for 4×44 k format one-color arrays. Images provided by the scanner were analyzed using Feature Extraction software v10.7.3.1 (Agilent Technologies). Raw signals were thresholded to 1 and quantile normalization [38] was performed using GeneSpring software. Data were considered in the log2 scale. Of the 41,105 probes present in the chip, 20,395 were suitable for further analysis because they fulfilled the following criteria: (i) they exhibited a signal within the higher 80th percentile in at least 75% of the replicates in one condition; (ii) at least 75% of the replicates in a given condition were flagged as present or marginal and (iii) the coefficient of variation across samples was larger than 2.5%. Quality control checks were based on the bioconductor package ArrayQualityMetrics (www.bioconductor.org). All samples employed for analysis were processed using the Limma bioconductor package [39]. For each contrast, genes with a Benjamini-Hochberg corrected p-value below 0.05 were considered to be differentially expressed [40]. Unless stated otherwise, each contrast focused on those genes that were differentially expressed and with a change of at least 2-fold between the compared conditions. The probe ID, gene names and log fold changes (logFC) of genes significantly expressed are listed in *supplementary material* (**Tables** **S1, S2, S3, S4, S5, and S6**). Functional analyses of the differentially expressed genes in the different contrasted conditions were obtained using the Ingenuity Pathway Analysis tool (www.ingenuity.com). Hierarchical and k-means clustering were performed on the normalized data using Genesis [41].

### Quantitative Real-time PCR

In order to validate microarray results, an independent analysis by qRT-PCR was performed for a subset of 22 genes (19 regulated genes plus 3 houskeeping genes). This selection was based on their FC values. To achieve this, a new cohort of 12 animals was used. Total RNA was quantified (ND-1000 Nanodrop) and evaluated for quality (2100 Bioanalyzer) before reverse transcription (High Capacity RNA to cDNA Master Kit) using 1 µg of total RNA in a 25 µl volume reaction. Real time PCR was performed using Taqman (Applied Biosystems) gene expression assays (GAPD, Rn01775763_g1; PGK1, Rn00821429_g1; HMBS, Rn00565886_m; BDNF, Rn02531967_s1; VEGFc, Rn01488076_m; OLIG2, Rn01767116_m; CASP8, Rn00574069_m; PCNA, Rn00574296_g1; LINGO3, Rn01408564_m; HSPA12a, Rn01410714_m; HES2, Rn00570311_g1; SEMA4f, Rn00570562_m; NEFM, Rn00566763_m; CXCL12, Rn00573260_m; CXCR4, Rn01483207_m; BMP4, Rn00432087_m; EGR1, Rn00561138_m; IGF1, Rn00710306_m; CALB1, Rn00583140_m; NRN1L, Rn01762396_g1; FGF18, Rn00433286_m; CXCL1, Rn00578225_m). Reactions were performed using reference genes GAPDH, PGK1 and HMBS in 384-well plates using 2.5 µL of total cDNA following the manufacturer’s instructions in a 7900 HT Fast Real-Time PCR System (Applied Biosystems) with SDS software and RQ Manager. All assays were done in triplicate.

## Supporting Information

Table S1 **Differentially expressed genes in the core at 24 h.**
(XLS)

Table S2 **Differentially expressed genes in the periinfarct at 24 h.**
(XLS)

Table S3 **Differentially expressed genes in the core at 3 d.**
(XLS)

Table S4 **Differentially expressed genes in the periinfarct at 3 d.**
(XLS)

Table S5 **Differentially expressed genes in the core compared with periinfarct at 24 h.**
(XLS)

Table S6 **Differentially expressed genes in the core compared with periinfarct at 3 d.**
(XLS)

## References

[1] Moskowitz MA, Lo EH, Iadecola C (2010) The science of stroke: mechanisms in search of treatments. Neuron 67: 181–198. 20670828 10.1016/j.neuron.2010.07.002 PMC2957363

[2] Zoghbi HY, Warren ST (2010) Neurogenetics: advancing the “next-generation” of brain research. Neuron 68: 165–173. 20955921 10.1016/j.neuron.2010.10.015 PMC2982747

[3] Cramer SC (2008) Repairing the human brain after stroke: I. Mechanisms of spontaneous recovery. Ann Neurol. 2008/04/03 ed. 272–287. 10.1002/ana.21393 18383072

[4] Cramer SC (2008) Repairing the human brain after stroke. II. Restorative therapies. Ann Neurol 63: 549–560. 18481291 10.1002/ana.21412

[5] Lo EH (2008) A new penumbra: transitioning from injury into repair after stroke. Nat Med 14: 497–500. 18463660 10.1038/nm1735

[6] Carmichael ST (2006) Cellular and molecular mechanisms of neural repair after stroke: making waves. Ann Neurol 59: 735–742. 16634041 10.1002/ana.20845

[7] Candelario-Jalil E (2009) Injury and repair mechanisms in ischemic stroke: considerations for the development of novel neurotherapeutics. Curr Opin Investig Drugs 10: 644–654. 19579170

[8] Gutierrez M, Merino JJ, de Lecinana MA, Diez-Tejedor E (2009) Cerebral protection, brain repair, plasticity and cell therapy in ischemic stroke. Cerebrovasc Dis 27 Suppl 1177–186. 19342849 10.1159/000200457

[9] Simon RP, Meller R, Zhou A, Henshall D (2012) Can genes modify stroke outcome and by what mechanisms? Stroke 43: 286–291. 22156698 10.1161/STROKEAHA.111.622225 PMC3282466

[10] Lu A, Tang Y, Ran R, Clark JF, Aronow BJ, et al (2003) Genomics of the periinfarction cortex after focal cerebral ischemia. J Cereb Blood Flow Metab 23: 786–810. 12843783 10.1097/01.WCB.0000062340.80057.06

[11] Hossmann KA (2008) Cerebral ischemia: models, methods and outcomes. Neuropharmacology 55: 257–70. 18222496 10.1016/j.neuropharm.2007.12.004

[12] Ramos-Cabrer P, Campos F, Sobrino T, Castillo J (2011) Targeting the ischemic penumbra. Stroke 42: S7–11. 21164112 10.1161/STROKEAHA.110.596684

[13] Foley LM, Hitchens TK, Barbe B, Zhang F, Ho C, et al (2010) Quantitative Temporal Profiles of Penumbra and Infarction During Permanent Middle Cerebral Artery Occlusion in Rats. Transl Stroke Res 1: 220–229. 21666857 10.1007/s12975-010-0032-6 PMC3109673

[14] Risher WC, Ard D, Yuan J, Kirov SA (2010) Recurrent spontaneous spreading depolarizations facilitate acute dendritic injury in the ischemic penumbra. J Neurosci 30: 9859–9868. 20660268 10.1523/JNEUROSCI.1917-10.2010 PMC2918261

[15] Sobrado M, Delgado M, Fernandez-Valle E, Garcia-Garcia L, Torres M, et al (2011) Longitudinal studies of ischemic penumbra by using 18F-FDG PET and MRI techniques in permanent and transient focal cerebral ischemia in rats. Neuroimage 57: 45–54. 21549205 10.1016/j.neuroimage.2011.04.045

[16] Kendziorski C, Irizarry RA, Chen KS, Haag JD, Gould MN (2005) On the utility of pooling biological samples in microarray experiments. Proc Natl Acad Sci U S A 102: 4252–4257. 15755808 10.1073/pnas.0500607102 PMC552978

[17] Zhan X, Ander BP, Jickling G, Turner R, Stamova B, et al (2010) Brief focal cerebral ischemia that simulates transient ischemic attacks in humans regulates gene expression in rat peripheral blood. J Cereb Blood Flow Metab 30: 110–118. 19738631 10.1038/jcbfm.2009.189 PMC2949112

[18] Marsh BJ, Williams-Karnesky RL, Stenzel-Poore MP (2009) Toll-like receptor signaling in endogenous neuroprotection and stroke. Neuroscience 158: 1007–1020. 18809468 10.1016/j.neuroscience.2008.07.067 PMC2674023

[19] Abe T, Shimamura M, Jackman K, Kurinami H, Anrather J, et al (2010) Key role of CD36 in Toll-like receptor 2 signaling in cerebral ischemia. Stroke 41: 898–904. 20360550 10.1161/STROKEAHA.109.572552 PMC2950279

[20] Ohab JJ, Fleming S, Blesch A, Carmichael ST (2006) A neurovascular niche for neurogenesis after stroke. J Neurosci 26: 13007–13016. 17167090 10.1523/JNEUROSCI.4323-06.2006 PMC6674957

[21] Cui X, Chen J, Zacharek A, Roberts C, Yang Y, et al (2009) Nitric Oxide Donor Upregulation of SDF1/CXCR4 and Ang1/Tie2 Promotes Neuroblast Cell Migration After Stroke. J Neurosci Res 87: 86–95. 18711749 10.1002/jnr.21836 PMC2606920

[22] Nagel S, Heinemann PV, Heiland S, Koziol J, Gardner H, et al (2011) Selective MMP-inhibition with Ro 28–2653 in acute experimental stroke–a magnetic resonance imaging efficacy study. Brain Res 1368: 264–270. 20971083 10.1016/j.brainres.2010.10.057

[23] Tanaka K, Nogawa S, Suzuki S, Dembo T, Kosakai A (2003) Upregulation of oligodendrocyte progenitor cells associated with restoration of mature oligodendrocytes and myelination in peri-infarct area in the rat brain. Brain Res 989: 172–179. 14556938 10.1016/s0006-8993(03)03317-1

[24] Espinosa de los Monteros A, de Vellis J (1988) Myelin basic protein and transferrin characterize different subpopulations of oligodendrocytes in rat primary glial cultures. J Neurosci Res 21: 181–187. 2464074 10.1002/jnr.490210210

[25] Cau E, Gradwohl G, Casarosa S, Kageyama R, Guillemot F (2000) Hes genes regulate sequential stages of neurogenesis in the olfactory epithelium. Development 127: 2323–2332. 10804175 10.1242/dev.127.11.2323

[26] Zhao X, He X, Han X, Yu Y, Ye F, et al (2010) MicroRNA-mediated control of oligodendrocyte differentiation. Neuron 65: 612–626. 20223198 10.1016/j.neuron.2010.02.018 PMC2855245

[27] De Val S, Black BL (2009) Transcriptional control of endothelial cell development. Dev Cell 16: 180–195. 19217421 10.1016/j.devcel.2009.01.014 PMC2728550

[28] Hayashi T, Abe K, Suzuki H, Itoyama Y (1997) Rapid induction of vascular endothelial growth factor gene expression after transient middle cerebral artery occlusion in rats. Stroke 28: 2039–2044. 9341716 10.1161/01.str.28.10.2039

[29] Weis SM, Cheresh DA (2005) Pathophysiological consequences of VEGF-induced vascular permeability. Nature 437: 497–504. 16177780 10.1038/nature03987

[30] Tang Y, Lu A, Aronow BJ, Wagner KR, Sharp FR (2002) Genomic responses of the brain to ischemic stroke, intracerebral haemorrhage, kainate seizures, hypoglycemia, and hypoxia. Eur J Neurosci 15: 1937–1952. 12099900 10.1046/j.1460-9568.2002.02030.x

[31] Bejot Y, Prigent-Tessier A, Cachia C, Giroud M, Mossiat C, et al (2011) Time-dependent contribution of non neuronal cells to BDNF production after ischemic stroke in rats. Neurochem Int 58: 102–111. 21074587 10.1016/j.neuint.2010.10.019

[32] Di Lazzaro V, Profice P, Pilato F, Dileone M, Florio L, et al (2007) BDNF plasma levels in acute stroke. Neurosci Lett 422: 128–130. 17590513 10.1016/j.neulet.2007.06.001

[33] Schönemeier B, Schulz S, Hoellt V, Stumm R (2008) Enhanced expression of the CXCl12/SDF-1 chemokine receptor CXCR7 after cerebral ischemia in the rat brain. J Neuroimmunol 198: 39–45. 18513805 10.1016/j.jneuroim.2008.04.010

[34] Robin AM, Zhang ZG, Wang L, Zhang RL, Katakowski M, et al (2006) Stromal cell-derived factor 1alpha mediates neural progenitor cell motility after focal cerebral ischemia. J Cereb Blood Flow Metab 26: 125–34. 15959456 10.1038/sj.jcbfm.9600172

[35] Popp A, Jaenisch N, Witte OW, Frahm C (2009) Identification of ischemic regions in a rat model of stroke. PLoS One 4: e4764. 19274095 10.1371/journal.pone.0004764 PMC2652027

[36] Broughton BR, Reutens DC, Sobey CG (2009) Apoptotic mechanisms after cerebral ischemia. Stroke 40: e331–339. 19182083 10.1161/STROKEAHA.108.531632

[37] Gutierrez-Fernandez M, Rodriguez-Frutos B, Alvarez-Grech J, Vallejo-Cremades MT, Exposito-Alcaide M, et al (2011) Functional recovery after hematic administration of allogenic mesenchymal stem cells in acute ischemic stroke in rats. Neuroscience 175: 394–405. 21144885 10.1016/j.neuroscience.2010.11.054

[38] Bolstad BM, Irizarry RA, Astrand M, Speed TP (2003) A comparison of normalization methods for high density oligonucleotide array data based on variance and bias. Bioinformatics 19: 185–193. 12538238 10.1093/bioinformatics/19.2.185

[39] Smyth GK (2004) Linear models and empirical bayes methods for assessing differential expression in microarray experiments. Stat Appl Genet Mol Biol 3: Article3. 16646809 10.2202/1544-6115.1027

[40] Benjamini Y, Drai D, Elmer G, Kafkafi N, Golani I (2001) Controlling the false discovery rate in behavior genetics research. Behav Brain Res 125: 279–284. 11682119 10.1016/s0166-4328(01)00297-2

[41] Sturn A, Quackenbush J, Trajanoski Z (2002) Genesis: cluster analysis of microarray data. Bioinformatics 18: 207–208. 11836235 10.1093/bioinformatics/18.1.207

